# Infectious Bovine Rhinotracheitis Control Program in Slovakia

**DOI:** 10.3389/fvets.2021.675521

**Published:** 2021-05-12

**Authors:** Rene Mandelik, Jozef Bires, Laszlo Ozsvari, Jaka Jakob Hodnik, Stefan Vilcek

**Affiliations:** ^1^Department of Epizootiology, Parasitology and Protection of One Health, University of Veterinary Medicine and Pharmacy, Košice, Slovakia; ^2^The State Veterinary and Food Administration of The Slovak Republic, Bratislava, Slovakia; ^3^Department of Veterinary Forensics and Economics, University of Veterinary Medicine, Budapest, Hungary; ^4^Clinic for Reproduction and Large Animals, Section for Ruminants, Veterinary Faculty, University of Ljubljana, Ljubljana, Slovenia

**Keywords:** IBR, control program, cattle, Slovakia, marker vaccine

## Abstract

As for other European countries, IBR is a significant cause of financial losses in cattle in Slovakia. The State Veterinary and Food Administration of the Slovak Republic prepared a voluntary IBR control program for cattle farms in 1995, which was implemented in 1996. In subsequent years, 48-119 farms/year enrolled in the voluntary IBR control program. Since the end of 2006, the IBR control program became compulsory by law for all cattle farms in Slovakia. Serology was used to identify infected animals using a conventional ELISA amongst non-vaccinated cattle and a gE specific ELISA in cattle vaccinated with marker vaccine. Eradication is based on culling when the serological prevalence of IBR in a herd is below 15%. When the prevalence is higher than 15%, the culling is combined with the application of a marker vaccine. A radical method where all animals are slaughtered is used with the agreement of the farmer when appropriate, especially for very small herds. Depending upon the selected eradication method, the antibody positive cattle can be gradually replaced in the herds to eliminate financial losses due to the disease. The movement of cattle is under strict control requiring a health certificate issued by the state veterinary authority and the movement must be recorded in the central livestock registry. The next step for herds is monitoring to achieve official IBR-free status. Based on the official figures from The State Veterinary and Food Administration, 60.2% herds were free of IBR in Slovakia in 2020.

## Introduction

Bovine herpes virus 1 (BoHV-1) is the causative agent of infectious bovine rhinotracheitis (IBR) and was first reported in dairy cattle in California 70 years ago. IBR was later diagnosed worldwide (1). In the 1950s, a new manifestation of BoHV-1 infection, infectious pustular vulvovaginitis (IPV), was described in cows and bulls. At present, IBR/IPV causes a wide range of clinical signs (including abortion, infertility, respiratory problems, encephalitis, conjunctivitis, enteritis, and dermatitis) due to inflammatory processes affecting the respiratory, genital and other organ systems (2). BoHV-1 may establish latency and virus can be shed intermittently (3). The triggering factors for shedding in latent infection, which is a potential source of BoHV-1 infection in the herd, may include cattle movement, unfavorable weather conditions, and poor husbandry or diet (3–5). Virus shedding at reactivation can be reduced but not eliminated by vaccination (6).

Big differences in seroprevalence and disease incidence were observed worldwide (5, 7). Veterinarians and farmers in Europe recognized the danger of BoHV-1 infection in cattle farms and started to implement control programs to eradicate IBR/IPV in several countries since the 1980s. All programs, voluntary or compulsory, were based on the removal of wild-type virus seropositive animals from the herds with or without the application of vaccination. Some European countries or regions are already declared as IBR-free, many others have introduced control programs^1^ (5).

As for other European countries, IBR/IPV can be a significant cause of financial loss due to respiratory and reproduction problems in Slovakian cattle. Virus infection has been detected by serology in all regions of Slovakia. However, clinical cases are rarely detected, for example, 14 cases with clinical signs of IBR were observed in 2003.^2^

The State Veterinary and Food Administration (SVFA) of the Slovak Republic has prepared an IBR control program (IBR CP) for cattle farms for all ages of animals in 1995, which was introduced the next year and has been continuously updated.^2^ The aim of this study is to summarize the basic principles of the IBR control program and its progress in Slovakian cattle farms. We also concentrate on specific problems of farmers in Slovakia with introduction of IBR CP on small and large farms.

## Materials and Methods

### Veterinary Organizations and Partners Involved in the CP

SVFA is the main organization in Slovakia dealing with all veterinary aspects and it is responsible for the IBR CP. There are 40 Regional Veterinary and Food Administration Offices responsible for the organization of the CP at the regional level. Of the four State Veterinary and Food Institutes with diagnostic laboratories, the State Veterinary Institute in Zvolen (central part of Slovakia) is the reference laboratory for IBR. All partners involved in the CP and their responsibilities are summarized in Table 1.

**Table 1:** Role of partners in IBR control program.

**Ministry of Agriculture and Rural Development of the Slovak Republic** • Approval of national plan for the CP • Decision on funding for the CP
**State Veterinary and Food Administration in the Slovak Republic** • Preparation of the CP and incorporation of important changes • Informing and educating the partners involved in the CP • Regular evaluation of progress of the CP • Preparation of the CP's economic plan for the ministry
**Regional Veterinary and Food Administrations** • Direct transfer of information to farmers • Education of veterinarians and farmers on the CP • Classification of herds • Evaluation of individual CP on a farm • Preparation of a report on the CP
**State Veterinary and Food Institutes** • Laboratory diagnosis of IBR • Qualified advice for Regional Veterinary and Food Administrations
**Reference laboratory for IBR** • Preparation of laboratory diagnostic method for the diagnosis of IBR • Qualified advice to other diagnostic laboratories
**Farmers** • Discuss and agree with the private veterinarian on the method used in the CP • Preparation of conditions for the introductory monitoring of the CP • Short reports on the running of the CP on a farm • Identification and registration of animals and transport of animals • Maintaining biosecurity measures, especially against reintroduction of infection

### Cattle, Herds, and the IBR CP

In February 2021, 451,257 cattle were registered in Slovakia. The animals were distributed in small (1-10 animals), medium (11-100 animals), and large farms (more than 101 animals). Density of cattle is 0.27 animal/ha of grass area (8).

The IBR CP prepared by SVFA was officially approved by the Ministry of Agriculture and Rural Development of the Slovak Republic and is published on its website^2^. The basic information on the IBR CP presented in this paper was taken from this document.

### Diagnostic Methods

Serological diagnosis of IBR/IPV is carried out in four diagnostic laboratories of the State Veterinary and Food Institutes. When seropositive herds are identified, all further laboratory analysis is carried out at the reference laboratory in Zvolen. The diagnosis of IBR/IPV is made using Ab-ELISA (IDEXX, Sweden) in samples from non-vaccinated cattle and Ab-gE-ELISA (IDEXX, Sweden) in samples from cattle vaccinated with a marker vaccine. In rare cases when clinical signs are observed, various methods are used for the detection of BoHV-1, such as Ag-ELISA (accredited in-house method), virus neutralization test,^3^ virus cultivation on MDBK and BT cell cultures^3^ and viral DNA detection with PCR (9).

### Eradication Methods Used in the CP

The herds involved in the CP have to be serologically screened for BoHV-1 specific antibodies to choose between available methods for IBR eradication on a farm.

Depending on the seroprevalence in the herd one of three methods are used in the CP:

(a) Elimination method combined with vaccination. This approach is used when the seroprevalence of IBR is over 15%. This threshold indicates more extensive infection requiring vaccination of the herd. Animals are vaccinated by a marker vaccine with a deleted glycoprotein, gE. Vaccination is not compulsory but highly recommended. Animals that are seropositive for the wild-type virus are gradually culled from the herds and replaced with new virus negative animals.
(b) Elimination method without vaccination. This method is used when the seroprevalence in the herd is under 15%. Seropositive animals are systematically eliminated from the herds as soon as possible and replaced with healthy serologically negative cattle.
(c) A radical method is used in the case of small herds where applying long-term systematic elimination methods is not economically sensible and a better solution for the farmer is the culling of the entire herd.

### Vaccination of Cattle

Inactivated and live vaccines against IBR are used in the IBR CP, according to the producers' instructions.

In the case of inactivated vaccines (Bovilis IBR marker inactivatum inj. susp., Intervet International B.V., Netherlands or Rispoval IBR marker inactivatum inj., Zoetis^®^, Czech Republic), the animals are first vaccinated when over 3 months old and revaccinated after 4 weeks. Subsequently revaccination is done after 6-months to maintain immunity. Administration of vaccine is i.m. (Bovilis) or s.c. (Rispoval).

When Bovilis live vaccine (Bovilis IBR marker live, Intervet International B.V.^®^, Netherlands) is used, the calves are vaccinated i.n. from 2 weeks to 3 months of age, the second dose being given i.m. at the age of 3-4 months, and subsequent revaccination after 6 months. If Rispoval live vaccine is used (Rispoval IBR marker vivum, Zoetis^®^, Czech Republic), the first dose is administrated i.n. to animals over 2 weeks in age, the second dose i.m., once animals are over 3 months, and then revaccination is after 6 months.

### Replacement of Cattle

Replacement of cattle in the recovery herd is under strict restrictions. All new animals for further breeding and production have to originate from officially IBR-free herds or IBR-free herds (see classification and definition of herds in Table 2) which are under state veterinary control. All animals from officially IBR-free herds older than 24 months must be confirmed serologically negative at 12 months intervals. The transferred animals can also originate from herds where cattle older than 6 months are vaccinated and regularly revaccinated with marker vaccine if they are intended for recovery herds. Replacement animals older than 6 months have to be serologically tested negative for antibodies against gE of BoHV-1 within the last 12 months and within the last 21 days before transfer to the recovery herd.

**Table 2:** Classification of the herds.

**Officially IBR-free herd** Definition: Herd with no BoHV-1 and no antibodies against wild-type virus and no antibodies after vaccination with a marker vaccine • No clinical signs of IBR/IPV were observed in the last 6 months • Herd has no contact with animals of lower IBR status • Insemination is under strict control with bull semen originating from officially IBR-free bull herds • Introductory and final monitoring for specific antibodies were negative • Herd is under control regime (monitoring)
**IBR-free herd** Definition: Herd with no BoHV-1 and no antibodies against wild-type virus but antibodies after vaccination with a marker vaccine can be present (recovered herd)
**Herd in recovery** • Herd with introductory screening • Herd running individual control (eradication) program
**Herd with unknown health status** • Herd with no screening and no data on IBR prevalence

### Monitoring

For monitoring of officially IBR-free herds, animals older than 9 months are sampled twice for serological testing at 5-7 months intervals. Subsequently, serological testing of all animals older than 24 months is performed at 12 months intervals. The monitoring of IBR-free herds is based on the analysis of five randomly selected animals older than 24 months from each stable to check for negative serological results. When samples are positive, further serological analysis or bulk tank milk (BTM) surveys on dairy farms continue. Confirmed positive farms must follow the procedures of the CP.

### Payment of Costs

Two partners bear the costs of the CP. SVFA pays for the initial screening, monitoring in recovered farms, final tests before ending of the recovery program, and final tests for detection of antibodies in farms recognized as officially IBR-free. The farmers pay for vaccination of animals and serological monitoring during the recovery program and costs of replacement animals.

## Results

### Flowchart of the CP on a Farm

**Figure 1:**
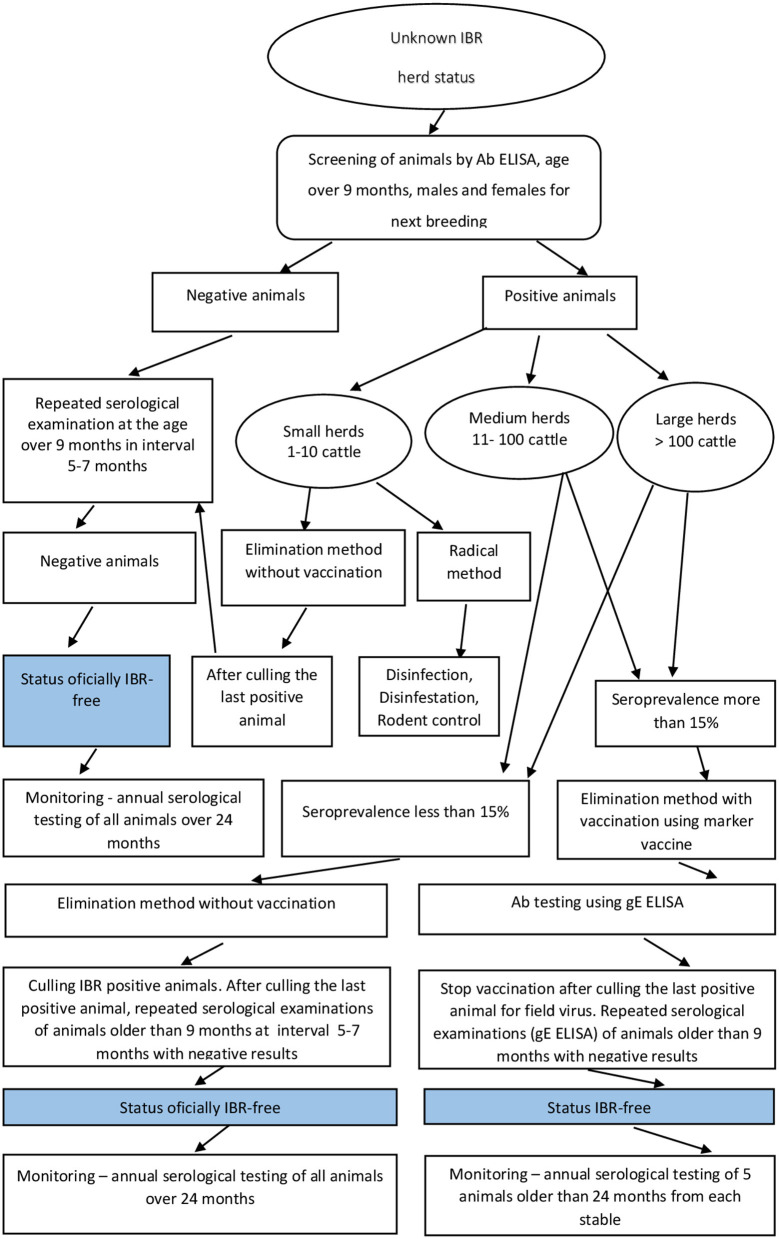
Overview flowchart of Infectious bovine rhinotracheitis control program in Slovakia.

An overview flowchart of IBR CP on the cattle farms in Slovakia is presented in Figure 1. A herd with specific antibodies detected is declared as infected and selects one of the three CP methods available (see M&M). The Regional Veterinary and Food Administration Office prepares a control mechanism for individual CP, within the frame of the official IBR CP. This will include a vaccination program, a plan for the replacement of animals, and an identification of the animal groups for serological monitoring, which are tested under the responsibility of mandated private veterinarians. Depending on the selected methods for eradication, the positive cattle are gradually culled and cattle are replaced by animals originating from officially IBR-free or IBR-free herds respecting the economic situation of the farmer. After replacement of all infected animals, the monitoring starts to maintain the classification as an IBR-free herd.

### Results of CP

At the start of the voluntary phase, 48 farmers implemented the program in 1996, 119 farmers joined the next year, and this number varied yearly but never reached more than a hundred farmers per year thereafter, until 2004.

Significantly more farmers implemented the CP when it became compulsory at the end 2006. Data on results of the IBR control program for the years 2000, 2013, 2019, and 2020 are summarized in Table 3. They indicate that despite two thirds to three quarters of the holdings being classified as recovered or involved in the recovery process, the remainder of the holdings, mostly small farms, with prepared individual CP still have to start and finish the program. When looking at large holdings only, all together 1,019 were registered in Slovakia till March, 2021. By the end of 2020, 625 large farms (61.3%) were free of IBR, 307 (30.1%) were in the recovery process and 57 (5.6%) were not tested yet.

**Table 3:** Numbers of holdings involved in IBR control program in Slovakia.

	**Officially IBR-free**			**IBR-free**			**In the recovery process**			**Not examined**		
	**Small**	**Medium**	**Large**	**Small**	**Medium**	**Large**	**Small**	**Medium**	**Large**	**Small**	**Medium**	**Large**
2010	3,950	552	129	15	28	53	311	342	907	2,314	142	6
2013	2,810	501	161	81	113	118	226	346	784	2,561	208	54
2019	3,001	563	197	93	101	413	195	330	345	1,869	185	52
2020	2,982	559	182	94	101	443	195	329	307	1,811	185	57

*Small holding: 1-10 animals, medium holding: 11-100 animals, large holding: 101 and more animals*.

Official figures from the SVFA from January 1, 2020 indicate that of 7,245 cattle farms, 3,723 farms (51.4%) were officially IBR-free, 638 farms (8.8%) were declared as IBR-free and 831 farms (11.5%) were in the recovery process. All together 60.2% farms were registered without IBR in Slovakia. However, at the beginning of 2020, 2,053 herds had yet to be tested (28.3%).

## Discussion

Many countries in Europe have applied IBR eradication programs based on different strategies (5), such as Scandinavian countries (10, 11), Switzerland (12), Germany (13), The Netherlands (14), Estonia (15), Hungary (16), and others. All the CPs are based on the removal of seropositive cattle from the herds with or without vaccination. Although live, attenuated or inactivated vaccines have been used in eradication of IBR (17–19), at present, the live and inactivated marker vaccines with deleted gE encoding genomes of BoHV-1 are used in these programs, as the marker vaccine helps to discriminate the infected animals within vaccinated herds (20).

Based on positive experiences in other European countries, the farmers in Slovakia also decided to introduce IBR CP in their farms. The basic aim of the CP was formulated as (i) to eradicate IBR/IPV in cattle farms, (ii) to improve the health status of animals, (iii) to decrease the losses in the cattle farm industry, (iv) to prevent eventual restrictions on internal and international trade of live cattle and their food commodities. The control program started as a voluntary project but farmers joined it too slowly. To achieve better progress, SVFA supported by state authorities, decided to change the voluntary program to one that is compulsory by law. This act has drastically changed the situation in control of IBR, leading to an approximately more than 10-fold increase in the number of holdings that joined the program each year. The progress of IBR eradication programs in Europe also indicates that compulsory programs are more effective than voluntary approaches (7, 14).

However, the progress in control of IBR in Slovakia has not been as fast as expected because the initial prediction was to finish the program in 7 years. Here, as in most other Eastern European countries with re-structured economies, the main problem with running the IBR CP is insufficient financial income and support for farmers due to economic problems in the country, especially in the agricultural sector. The costs for running the control program, i.e., price for laboratory investigation, vaccination, replacement of animals are too high for farmers. Despite IBR CP being compulsory and farms receiving customized IBR CPs, the economically weaker farms have problems to follow all the rules of the program.

When analyzing the IBR CP in Slovakia we see different motivation of farmers with small and large holdings. Most farms are very small with not more than 10 animals, 90% of them house 1-2 animals only. Production of these farms is focused for individual consumption of food products by the farming family and production of cattle dung. These farmers have no strong motivation to join the CP and bear the program costs. In their experience, when cattle are negative for IBR, the animals remain healthy for a long time as new cattle that might introduce infection are rarely introduced. In infected farms, the replacement of animals is rather slow. Vaccination is not welcomed without visible additional production value. However, despite the slow recovery process, the numbers of infected animals in small farms have diminished due to gradual recovery of commercial farms which are the main source for new animals bought by small farmers.

On the other hand, farmers with larger farms are more motivated to join the CP. They expect and usually achieve better health status, higher reproduction indicators and lower numbers of abortions and mortality rate in their herds. Export of animals is an additional incentive for farmers to join the program, but avoidance of restrictions on international trade was less important for those that do not export cattle or their food commodities.

It is logical to ask why some farms have been more successful with the CP than others. A critical analysis revealed several factors. The progress with CP depends not only on financial support for the program, which is, of course, very important, but also on education of farmers about animal health, the organization of work on the farm, the coordination of the program by the regional veterinary offices and the local level of veterinary health care for cattle. These factors vary from farm to farm and significantly influence the running of the CP.

Education about the CP for small farmers is carried out by private veterinarians who provide advice on the diagnosis and control of IBR (and other diseases), make recommendations on vaccination, and provide details on customized CP options. Farmers with medium and large holdings are better informed about the national CP by the farmer union organization and through the regional veterinary offices and by education from workshops and conferences focused on virus transmission, clinical signs, reproductive problems due to virus infection etc.

The reintroduction of infection is a big danger for recovered or IBR-free herds. The main risk factors for disease reintroduction are the purchase of animals, direct contact between different herds, especially with those of unknown status, and *via* contaminated semen (21–24). Reintroduction of infection has been recorded by OIE Reports in several IBR-free countries, such as Austria, Denmark, and Switzerland (5). Despite strict conditions for movement of animals for herds involved in the IBR CP in Slovakia, reinfection has been observed where more than one farm is owned by the same owner, usually co-located in a common region. The recovery process in these farms was not synchronized, including vaccination and replacement of animals, providing an opportunity for direct and indirect contacts between animals of differing health status, e.g., through animal movements or uncontrolled traffic and common personnel. In some cases, despite vaccination having been completed, the reintroduction of infection was observed in herds in several months. Similar mistakes were observed in fattening herds. Again, the reinfection occurred due to uncontrolled mixing and movement of animals between vaccinated and infected cattle.

The experiences of farmers with the IBR CP in Slovakia can provide some recommendations for farmers in other countries considering similar programs. If possible, the CP should be compulsory with significant financial support from the government or other commercial partners. Special attention should be paid not only to big farms but particularly to small farms where motivation to participate in joint programs is usually low or negative. Attention should also be paid for harmonization of work on different farms and by partners involved in the CP to ensure that best practices are followed uniformly at least at regional level but better still at national level. The control of movement of animals, especially between farms under common ownership, is essential to prevent uncontrolled mixing of herds with different health status.

The successful eradication and attainment of official IBR-free status has already been achieved in Scandinavian countries (Denmark, Finland, Norway, and Sweden), Switzerland, Germany, Province Bolzano and Valle d'Aosta in Italy and on the island of Jersey in the UK.^4^ Of countries surrounding Slovakia, Austria is already an IBR-free country. The Czech Republic obtained the status of an IBR-free country in November 2020.^4^ Hungary, Ukraine, and Poland also run IBR CPs.^5^ The IBR CP is a big challenge for the Slovakian farmers and the program runs more progressively in large farms than in small herds. The farms involved in international trade are naturally forced to have an approved eradication program or to become IBR-free to benefit from additional EU guarantees for cattle trade according to articles 9 and 10 of the EU Directive 64/432/EEC, respectively.

In conclusion, the IBR CP in Slovakia is in progress with specific problems, especially in small farms, where the program runs slowly. Despite the complicated economic situation in the country, which significantly influences the running of the program, the successful recovery of most large holdings provides encouragement that the IBR CP in Slovakia can be finished in a short time.

## Data Availability Statement

The raw data supporting the conclusions of this article will be made available by the authors, without undue reservation.

## Ethics Statement

Ethical review and approval was not required for the animal study because results were taken from national IBR control program approved by The State Veterinary and Food Administration of the Slovak Republic. Written informed consent for participation was not obtained from the owners because results of this study are a part of official national IBR control program.

## Author Contributions

RM and JB collected the results of IBR CP. RM and SV wrote the manuscript. JH and LO contributed to the evaluation of results and edited the text of manuscript. All authors read and approved the submitted version.

## Conflict of Interest

The authors declare that the research was conducted in the absence of any commercial or financial relationships that could be construed as a potential conflict of interest.

## Footnotes

^1^Commission Decision 2004/558/EC, Commission implementing decision (EU) 2020.

^2^Plan eradikacie infekcnej bovinnej rinotracheitidy (IBR) na Slovensku na rok 2020 (in Slovak language) (2020): https://www.svps.sk/dokumenty/zvierata/2020/1554_001.pdf (Accessed January 28, 2021).

^3^OIE Terrestrial Manual 2010.

^4^Provadeci rozhodnuti komise (EU) 2020/1663 ze dne 6. listopadu 2020 (2020): https://eur-lex.europa.eu/legal-content/CS/TXT/PDF/?uri=CELEX:32020D1663&from=CS (Accessed January 28, 2021).

^5^COST Action [CA17110], Deliverable 1.1.
